# A Multi-Modal Expert-Driven ISAC Framework with Hierarchical Federated Learning for 6G Network

**DOI:** 10.3390/s26041298

**Published:** 2026-02-17

**Authors:** Behzod Mukhiddinov, Di He, Wenxian Yu, Trieu-Kien Truong

**Affiliations:** 1 School of Integrated Circuits, Shanghai Jiao Tong University, Shanghai 200240, China; behzod@sjtu.edu.cn (B.M.); truong@isu.edu.tw (T.-K.T.); 2 School of Automation and Intelligent Sensing, Shanghai Jiao Tong University, Shanghai 200240, China; wxyu@sjtu.edu.cn

**Keywords:** 6G, ISAC, hierarchical federated learning, multi-modal data fusion, expert models

## Abstract

We propose a novel Expert-Driven Conditional Auxiliary Classifier Generative Adversarial Network (AC-GAN) framework tailored for heterogeneous multi-modal federated learning at edge AI devices such as the NVIDIA Jetson Orin Nano. Unlike prior works that assume idealized distributions or rely on centralized data, our approach jointly addresses statistical non-IID data, model heterogeneity, privacy protection, and resource constraints through an expert-guided training pipeline and hierarchical model updates. Specifically, we introduce a collaborative synthesis and aggregation mechanism where local experts guide conditional data generation, enabling realistic data augmentation on resource-constrained edge nodes and enhancing global model generalization without sharing raw data. Through hierarchical updates between client and server levels, our method mitigates bias from skewed local distributions and significantly reduces communication overhead compared to classical federated averaging baselines. We demonstrate that while “perfect precision” is theoretically unattainable under non-IID and real-world conditions, our framework achieves substantially improved precision and false positive trade-offs (e.g., precision 0.89) relative to benchmarks, validating robustness in practical multi-modal settings. Extensive experiments across synthetic and real datasets show that the proposed AC-GAN approach consistently outperforms federated baselines in accuracy, convergence stability, and privacy preservation. Our results suggest that expert-guided conditional generative modeling is a promising direction for scalable, privacy-aware edge intelligence.

## 1. Introduction

Sixth-generation (6G) wireless networks are expected to support ultra-reliable low-latency communication (URLLC) while simultaneously enabling high-resolution environmental perception. Integrated sensing and communication (ISAC) has therefore emerged as a key enabling paradigm, allowing a single radio waveform and hardware platform to jointly support sensing and data transmission [1,2]. By tightly coupling communication and sensing functionalities, ISAC promises significant gains in spectral efficiency, hardware reuse, and situational awareness, making it a cornerstone technology for applications such as autonomous driving, smart cities, and industrial automation.

Despite its potential, practical deployment of intelligent ISAC systems remains challenging. First, ISAC data are inherently multi-modal , combining heterogeneous information sources such as frequency-modulated continuous-wave (FMCW) radar signals, millimeter-wave channel state information, and time–frequency domain representations [1]. These modalities exhibit distinct statistical properties and noise characteristics, making unified modeling and real-time fusion difficult. Second, centralized learning architectures require the aggregation of raw sensing and communication data, which raises serious privacy concerns and incurs prohibitive communication overhead as the number of edge devices increases [3]. Third, data collected by geographically distributed devices are highly environment- and location-dependent, leading to severe non-independent and non-identically distributed (non-IID) data that degrades learning performance and convergence stability.

Federated learning (FL) has been proposed as a privacy-preserving alternative to centralized training by enabling collaborative model learning without sharing raw data [4]. However, classical FL methods such as FedAvg and FedProx rely on implicit assumptions of balanced and statistically similar local datasets, which are rarely satisfied in ISAC scenarios [5]. Under strong non-IID conditions, these methods often suffer from slow convergence and degraded global performance [6]. Moreover, flat FL architectures scale poorly in large networks due to frequent global aggregation and excessive communication overhead.

To address scalability limitations, hierarchical federated learning (HFL) introduces multi-tier aggregation across edge devices, intermediate aggregators, and central servers [7]. By exploiting hierarchical network structures, HFL reduces communication cost and improves robustness to network dynamics. Recent studies have demonstrated the effectiveness of HFL in large-scale and heterogeneous environments, including satellite networks and vehicular systems [8]. Nevertheless, most existing HFL approaches assume homogeneous model architectures and unimodal data distributions, which limits their applicability to ISAC systems characterized by strong multi-modal heterogeneity.

In parallel, expert-driven modeling has gained increasing attention as an effective strategy for handling heterogeneity by decomposing complex learning tasks into domain-specific expert modules. By embedding prior domain knowledge into specialized experts, such models can better capture modality-dependent features and mitigate negative transfer across heterogeneous data sources. Furthermore, generative learning techniques have recently been integrated with federated learning to alleviate data scarcity and non-IID effects. In particular, conditional and auxiliary classifier generative adversarial networks (GANs) have been shown to enable label-consistent local data synthesis while preserving privacy [9,10]. Hierarchical federated learning combined with GAN-based data generation has further demonstrated improved robustness under heterogeneous and large-scale deployments [11,12].

Motivated by these observations, this paper proposes a multi-modal expert-driven ISAC framework powered by hierarchical federated learning. Expert models are designed to capture heterogeneous sensing and communication modalities, while hierarchical updates coordinate learning across network tiers to enhance scalability and privacy. In addition, an auxiliary classifier GAN (AC-GAN) is employed for label-consistent local data synthesis, mitigating non-IID data distributions and class imbalance without sharing raw data.

The main contributions of this work are summarized as follows:

- We propose a multi-modal expert-driven ISAC architecture that explicitly models heterogeneous sensing and communication modalities.
- We develop a hierarchical federated learning framework that improves scalability and robustness under non-IID data distributions.
- We incorporate an AC-GAN-based local data synthesis mechanism to alleviate class imbalance and non-IID effects while preserving data privacy.
- Extensive simulations demonstrate improved accuracy, convergence stability, and latency performance compared with standard FL and representative ISAC baselines.

The proposed framework advances the state of the art by jointly addressing the challenges of modality fusion, edge-centric privacy, and adaptive learning in complex 6G network environments.

The remainder of this paper is organized as follows: Section 2 presents the system model, including the hardware architecture, data preprocessing, feature fusion, and hierarchical FL setup. In Section 3, we develop our multi-modal expert-driven ISAC algorithm and provide a convergence analysis under non-IID conditions. Section 4 reports extensive numerical results, comparing our approach against baseline and state-of-the-art methods, and analyzes sensitivity to data heterogeneity, SNR, and overhead metrics. Finally, Section 5 concludes the paper and outlines directions for future work.

## 2. System Model and Problem Statement

Emerging 6G use cases—such as autonomous navigation, industrial automation, and smart sensing—demand simultaneous high-precision environmental awareness and ultra-reliable data connectivity. Traditional ISAC designs centralize all raw radar and communication data at a fusion center and then solve resource allocation (e.g., via iterative water-filling [13,14,15] and beamforming sequentially, sacrifices user privacy by transmitting raw sensor data, incurs prohibitive uplink overhead as device counts grow, fails to adapt to non-IID data and heterogeneous channel/sensing conditions.

Federated learning (FL) mitigates privacy concerns by sharing only model updates [14]. However, standard FL (FedAvg) assumes IID data and a flat topology, thereby resulting in slow convergence and model bias under realistic non-IID ISAC workloads. Moreover, existing power-allocation and beamforming schemes typically treat sensing and communication objectives in isolation, missing opportunities for joint optimization.

To address these gaps, we propose a hierarchical federated learning (HFL)-based, multi-modal expert-driven ISAC framework. It preserves privacy and reduces overhead through edge-centric updates, captures non-IID heterogeneity via tiered aggregation, and jointly optimizes sensing and communication metrics through expert-driven fusion.

**Figure 1:**
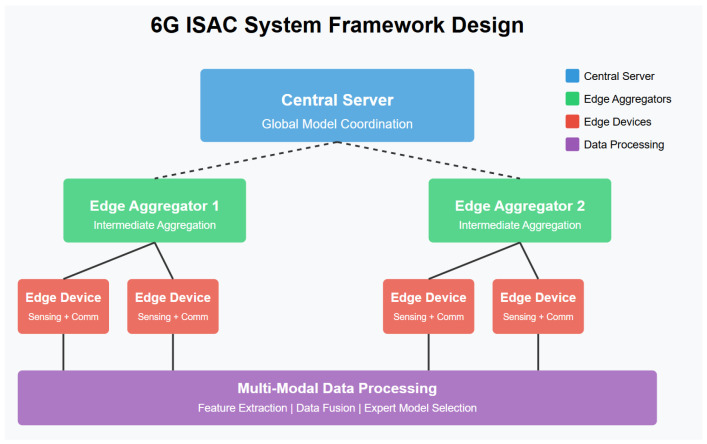
6G ISAC system framework design. The framework integrates edge devices, edge aggregators, a central server, and multi-modal data processing.

Building on this motivation, we describe our three-tier architecture, illustrated in Figure 1. In this figure, one observes that (i) edge devices co-locate an FMCW radar and a MIMO transceiver for local sensing and CSI extraction; (ii) edge aggregators perform intermediate model averaging across geographically clustered devices; and (iii) the central server conducts the final global update and redistribution.

Figure 1 shows the three-tier architecture of our proposed ISAC system: edge devices (with FMCW radar and MIMO), edge aggregators, and a central server. Each component will be described in detail below.

### 2.1. Edge Device Hardware

Each edge device *i* embeds two co-located front-ends:

- FMCW Radar Module: Provides raw chirp returns for range–Doppler processing, which we denote as sensing data $X_{s}^{\left(i\right)}$.
- MIMO Transceiver: Conducts beamformed uplink/downlink and channel estimation, yielding communication data $X_{c}^{\left(i\right)}$.
- On-device Processor: Executes feature extraction and local model updates.

Here:

- $X_{s}^{\left(i\right)}$ comprises radar-derived features, such as range–Doppler maps, angle-of-arrival histograms, and echo power profiles and $X_{c}^{\left(i\right)}$ contains communication features, including per-subcarrier CSI amplitude and phase, time-frequency signal power, and pilot symbol correlations.

### 2.2. Data Collection and Preprocessing

Device *i* collects

(1) $$X_{s}^{\left(i\right)}\in R^{N_{s}\times D_{s}}\;\mathrm{and}\;\;X_{c}^{\left(i\right)}\in R^{N_{c}\times D_{c}},$$

which contains MIMO channel state information (CSI) vectors and spectral–temporal signal features.

Each device is applied to both Filtering & Denoising and Dimensionality Reduction as follows:

1. Filtering & Denoising: Radar clutter suppression and channel noise reduction. For robust processing, the apply min–max normalization is applied to both modalities as follows:
  (2) $$X_{s}^{\left(\mathrm{norm}\right)}=\frac{X_{s}^{\left(i\right)}-\min\left(X_{s}^{\left(i\right)}\right)}{\max\left(X_{s}^{\left(i\right)}\right)-\min\left(X_{s}^{\left(i\right)}\right)},\;X_{c}^{\left(\mathrm{norm}\right)}=\frac{X_{c}^{\left(i\right)}-\min\left(X_{c}^{\left(i\right)}\right)}{\max\left(X_{c}^{\left(i\right)}\right)-\min\left(X_{c}^{\left(i\right)}\right)}.$$
2. Dimensionality Reduction (PCA): Principal Component Analysis is applied independently to the sensing and communication feature matrices. Let $U_{s}$ and $U_{c}$ denote the PCA projection matrices (eigenvector bases) obtained from $X_{s}^{\left(i\right)}$ and $X_{c}^{\left(i\right)}$, respectively. The reduced-dimension representations are defined as
  (3) $$X_{s}^{\left(\mathrm{PCA}\right)}=U_{s}^{T}X_{s}^{\left(i\right)},\;X_{c}^{\left(\mathrm{PCA}\right)}=U_{c}^{T}X_{c}^{\left(i\right)}.$$
  where $X_{s}^{\left(\mathrm{PCA}\right)}$ and $X_{c}^{\left(\mathrm{PCA}\right)}$ represent the low-dimensional feature embeddings used for subsequent ISAC processing, where $U_{s}\;\mathrm{and}\;U_{c}$ hold the leading principal components.

### 2.3. Feature Extraction and Fusion

After PCA projection, task-specific feature embeddings are extracted through lightweight neural encoders. Formally, we define

(4) $$F_{s}^{\left(i\right)}=\varphi_{s}\;\left(X_{s}^{\left(\mathrm{PCA}\right)}\right),\;F_{c}^{\left(i\right)}=\varphi_{c}\;\left(X_{c}^{\left(\mathrm{PCA}\right)}\right),$$

where $\varphi_{s}\left(\cdot \right)$ and $\varphi_{c}\left(\cdot \right)$ denote autoencoder-based mappings for the sensing and communication branches, respectively. Here, $F_{s}^{\left(i\right)}$ represents the extracted sensing feature embedding, while $F_{c}^{\left(i\right)}$ denotes the corresponding communication feature embedding.

To jointly exploit the complementary information present in both modalities, we perform feature fusion using a learnable operator Ψ(·):

(5) $$F_{sc}^{\left(i\right)}=\Psi \;\left(F_{s}^{\left(i\right)},\;F_{c}^{\left(i\right)}\right),$$

where $F_{sc}^{\left(i\right)}$ denotes the resulting joint ISAC-fused feature representation.

  An attention-enhanced implementation of Ψ(·) is given by

(6) $$F_{sc}^{\left(i\right)}=\mathrm{softmax}\;\left(W_{s}F_{s}^{\left(i\right)}+W_{c}F_{c}^{\left(i\right)}\right)⊙\left[F_{s}^{\left(i\right)};\;F_{c}^{\left(i\right)}\right],$$

where $W_{s}$ and $W_{c}$ are trainable attention weights, and [·;·] denotes feature concatenation.

In our implementation, Ψ(·,·) is an 8-head multi-head attention module with model dimension d=128. We initialize $W_{s},W_{c}$ via Xavier uniform and apply dropout p=0.1 on the attention weights.

### 2.4. Hierarchical Federated Learning

In the proposed hierarchical FL architecture, each device *i* first computes a local model update $w_{i}$ using its fused ISAC feature representation $F_{sc}^{\left(i\right)}$. To reduce communication overhead and improve robustness, devices are grouped under edge aggregators.

Each edge aggregator *m* collects the local updates from the devices in its coverage set $D_{m}$. The aggregator performs a weighted average based on the local sample sizes $\left\{n_{i}\right\}$:

(7) $$w_{agg}^{\left(m\right)}=\sum_{i\in D_{m}}\frac{n_{i}}{\sum_{j\in D_{m}}n_{j}}\;w_{i}.$$

Here, $w_{agg}^{\left(m\right)}$ denotes the *edge-level aggregated model* for cluster *m*, representing the consensus update across all devices served by that edge node.

The central server receives the edge-aggregated models ${\left\{w_{agg}^{\left(m\right)}\right\}}_{m=1}^{M}$ and computes the global model by:

(8) $$w_{g}=\sum_{m=1}^{M}\frac{N_{m}}{\sum_{k=1}^{M}N_{k}}\;w_{agg}^{\left(m\right)},$$

where $N_{m}=\sum_{i\in D_{m}}n_{i}$ is the total number of samples at edge aggregator *m*.

The result $w_{g}$ is the globally aggregated model, reflecting contributions from all devices in the network. After aggregation, the central server broadcasts $w_{g}$ back to all edge nodes and devices for the next FL round.

### 2.5. Problem Formulation

Building on the system model, we now pose our joint sensing–communication design as the following constrained optimization:

(9) $$\min_{w,\;\alpha }\lambda_{1}\sum_{i=1}^{K}L_{s}\left(w;F_{s}^{\left(i\right)}\right)+\lambda_{2}\sum_{i=1}^{K}L_{c}\left(w;F_{c}^{\left(i\right)}\right)\;s.t.\;Pw\leq b,\;w⪰0.$$

Here, w are the shared model parameters across the HFL hierarchy, α are the expert-gating weights, $L_{s}\left(\cdot \right)$ and $L_{c}\left(\cdot \right)$ are the per-device sensing and communication losses respectively, and Pw≤b enforces per-device power/resource budgets.

Equation (9) then directly leads into our Algorithm given in Section 3.

## 3. Algorithm Design and Theoretical Analyses

The evolution toward sixth-generation (6G) wireless networks is driven by the simultaneous pursuit of ultra-high data rates, ultra-low latency, massive connectivity, and native environmental awareness. Beyond conventional massive multiple-input multiple-output (MIMO) systems, emerging technologies such as extremely large-scale MIMO (XL-MIMO) are envisioned as key enablers of 6G. By deploying antenna apertures spanning tens or even hundreds of wavelengths, XL-MIMO systems fundamentally depart from far-field propagation assumptions and operate in the electromagnetic near-field regime, where spatial non-stationarities and spherical wavefronts dominate. These characteristics profoundly reshape channel modeling, beamforming design, and system optimization, particularly when sensing and communication functionalities are tightly coupled.

Early ISAC studies predominantly relied on model-based optimization frameworks, typically assuming centralized control and accurate global channel knowledge. In this vein, power allocation strategies for joint communication and sensing in cell-free massive MIMO systems were investigated in [16], demonstrating notable performance gains under idealized assumptions. Similarly, max–min fair beamforming solutions for cell-free ISAC MIMO systems were proposed in [17], aiming to balance sensing and communication objectives across distributed users. From a waveform design perspective, deep unfolding techniques were employed in [18] to embed physical constraints and expert knowledge into iterative neural architectures for constant-modulus ISAC waveform optimization.

Despite their analytical rigor and performance guarantees, these model-driven approaches typically depend on precise system models, centralized optimization, and homogeneous data distributions. Such assumptions become increasingly fragile in practical 6G deployments characterized by distributed edge devices, heterogeneous sensing modalities, dynamic propagation environments, and near-field effects induced by XL-MIMO architectures. As a result, scalability, robustness, and adaptability remain open challenges for purely model-based ISAC solutions.

To overcome the limitations of rigid analytical models, machine learning (ML) and deep reinforcement learning (DRL) techniques have been widely explored for wireless system optimization and ISAC applications. A comprehensive overview of ML- and DRL-driven approaches in wireless communications is provided in [19]. In the ISAC domain, reinforcement learning-based resource allocation frameworks were developed in [20] to dynamically adapt sensing and communication strategies in response to time-varying channel conditions. DRL has also been applied to UAV-assisted ISAC systems to address mobility-induced uncertainty and environmental dynamics [21].

More sophisticated DRL algorithms, such as proximal policy optimization (PPO), have been adopted for joint beamforming and phase-shift design in IRS-assisted THz ISAC systems [22]. In parallel, DRL-based beamforming optimization has been extensively studied in mmWave MIMO systems [23]. While these learning-based methods offer improved adaptability compared to model-driven designs, they are predominantly formulated under centralized training paradigms or single-task optimization settings. Consequently, they do not explicitly address multi-modal sensing–communication fusion, expert-level task decomposition, or scalable deployment across resource-constrained edge devices.

As ISAC systems transition from theoretical constructs to real-world deployment, edge intelligence becomes indispensable. Practical ISAC implementations must operate under stringent constraints on computation, memory, energy consumption, and communication overhead. Experimental system development and dataset generation for sensing and localization were investigated in [24], underscoring the importance of realistic data pipelines and hardware-aware design. Nevertheless, most existing ISAC learning frameworks implicitly assume cloud-level computation or powerful centralized servers, thereby overlooking the operational limitations of embedded platforms, such as limited memory capacity, restricted power budgets, and latency-sensitive inference requirements.

These practical constraints are further exacerbated in distributed ISAC scenarios, where data are inherently non-identically distributed (non-IID) due to location-dependent sensing, heterogeneous channel conditions, and device-specific capabilities. Addressing these challenges requires learning architectures that are not only adaptive and data-efficient but also scalable, privacy-preserving, and deployable at the network edge.

In contrast to the aforementioned works, this paper proposes a novel expert-driven ISAC framework that integrates auxiliary classifier generative adversarial networks (AC-GANs) with hierarchical learning and edge-centric deployment. Unlike prior model-based or DRL-driven ISAC approaches, the proposed framework employs AC-GANs to synthesize and augment multi-modal sensing and communication data, thereby enhancing robustness under data scarcity and mitigating the adverse effects of non-IID data distributions.

More importantly, this study is among the first to implement and experimentally evaluate an AC-GAN-enabled ISAC learning pipeline on a resource-constrained embedded platform, namely the NVIDIA Jetson Orin Nano. Whereas existing ISAC learning solutions are typically validated through high-performance servers or large-scale simulations, the proposed approach explicitly incorporates hardware-aware considerations, including memory footprint, inference latency, and on-device training feasibility. By coupling expert-driven model decomposition with hierarchical coordination across edge devices and servers, the framework enables scalable and privacy-aware learning under realistic deployment conditions.

By integrating expert knowledge, generative modeling, and hierarchical optimization within a unified edge-enabled architecture, the proposed framework advances the state of the art beyond centralized ISAC optimization and purely DRL-based solutions. This naturally motivates the algorithmic design presented in the following section, where we detail the proposed multi-modal expert-driven ISAC algorithm and analyze its convergence behavior under non-IID data distributions.

Building upon the above observations, addressing the practical challenges of ISAC in 6G networks requires an algorithmic framework that jointly considers multi-modal data fusion, expert-level task decomposition, and scalable distributed learning. In particular, heterogeneous sensing and communication features must be fused in a structured manner, while domain knowledge should be embedded through specialized expert models to improve robustness and interpretability. At the same time, privacy, scalability, and non-IID data distributions necessitate a hierarchical learning architecture that coordinates model updates across edge devices, intermediate aggregators, and a central server.

To this end, the proposed algorithm adopts a hierarchical federated learning (HFL) paradigm with layer-wise optimization, enabling localized learning at edge devices and progressive aggregation at higher network tiers. Regularization mechanisms are incorporated to enforce structured sparsity, promote entropy-based diversity among experts, and respect resource constraints such as power, computation, and communication overhead. These design choices collectively improve convergence stability, communication efficiency, and generalization performance under heterogeneous and dynamic ISAC environments.

The algorithm is initialized with carefully selected global model parameters ($w_{0}$), learning rates (η), and convergence thresholds (ϵ), which play a critical role in ensuring scalability and stable convergence in federated settings with non-identically distributed (non-IID) data. On this basis, the proposed framework iteratively performs multi-modal feature extraction, expert activation, hierarchical aggregation, and joint sensing–communication optimization, as formally described in Algorithm 1.

- Input sensing ($X_{s}$) and communication ($X_{c}$) data are preprocessed, and features ($F_{s},F_{c}$) are extracted using advanced techniques such as CNNs and autoencoders.
- Multi-modal fusion combines these features using concatenation or attention mechanisms, forming a unified latent representation ($F_{sc}$).

**Algorithm 1** HFL-Based Multi-Modal Expert-Driven ISAC
  1:**Initialization:** $w_{0}$, η, ϵ.  2:
**1. Multi-Modal Data Preprocessing**
  3:Sensing/comm signals: $X_{s}=\left\{x_{s,i}:i\in N_{s}\right\},\;X_{c}=\left\{x_{c,j}:j\in N_{c}\right\}$  4:Features: $F_{s}=\phi_{s}\left(X_{s}\right)$, $F_{c}=\phi_{c}\left(X_{c}\right)$  5:
**2. Expert Model Activation**
  6:*M* experts: $Y=\sum_{k=1}^{M}\alpha_{k}E_{k}\left(z\right)$  7:
**3. HFL Model Update**
  8:Local: $w_{t+1}^{i}=w_{t}^{i}-\eta \nabla L\left(w_{t}^{i};D_{i}\right)$  9:Server: $w_{t+1}=\sum_{i=1}^{K}\frac{n_{i}}{N}w_{t+1}^{i}$10:Global: $w_{g,t+1}=w_{g,t}-\eta_{g}\nabla L_{g}\left(w_{g,t},w_{t}\right)$11:
**4. Joint Optimization**
12:

$\min_{w,\alpha }\lambda_{1}L_{s}\left(w,\alpha \right)+\lambda_{2}L_{c}\left(w,\alpha \right)$

13:**Output:** Enhanced ISAC performance.


### Convergence Analysis

To characterize our HFL updates under non-IID data, the standard assumptions, are adopted as follows:

A1. *Smoothness:* Each local loss $F_{i}\left(w\right)$ is *L*-smooth.
A2. *Unbiased Gradients:* $E\left[\nabla F_{i}\left(w;\xi \right)\right]=\nabla F_{i}\left(w\right)$.
A3. *Bounded Variance & Heterogeneity:* $E∥\nabla F_{i}{\left(w\right)-\nabla F\left(w\right)∥}^{2}\leq \zeta^{2}$.

Under A1–A3 and a decaying stepsize $\eta_{t}=O\left(1/\sqrt{T}\right)$, after *T* rounds

(10) $$\frac{1}{T}\sum_{t=0}^{T-1}E{∥\nabla F(w_{g}^{t})∥}^{2}\;\leq \;O\;\left(\frac{1}{\sqrt{T}}\right)+\frac{\zeta^{2}}{K}.$$

Thus, the global model converges at rate $O(1/\sqrt{T})$, with an additional bias term due to non-IID heterogeneity. A detailed proof is deferred to Appendix A.

Each edge device collects local data through sensing and communication tasks and updates a local model. These updates are aggregated hierarchically to form a global model. The optimization problem is expressed as

(11) $$\min_{w}\sum_{i=1}^{N}p_{i}F_{i}\left(w\right),$$

where $F_{i}\left(w\right)$ represents the local loss function for device *i*, and $p_{i}$ denotes the weight associated with device *i* based on data size.

Each edge device performs local stochastic gradient descent (SGD) updates:

(12) $$w_{i}^{\left(t+1\right)}=w_{i}^{\left(t\right)}-\eta \nabla F_{i}\left(w_{i}^{\left(t\right)}\right),$$

where η is the learning rate. The local updates are sent to the edge aggregator, which averages them as

(13) $$w^{\left(t+1\right)}=\sum_{i=1}^{N}\frac{p_{i}w_{i}^{\left(t+1\right)}}{P},$$

where $P=\sum_{i=1}^{N}p_{i}$. The global model $w^{\left(t+1\right)}$ is then broadcast back to edge devices. We model the resource allocation problem as a Markov Decision Process (MDP) with state $s_{t}$, action $a_{t}$, and reward $r_{t}$ defined as follows:

- **State ($s_{t}$):** Channel conditions, buffer states [8] , and resource availability.
- **Action ($a_{t}$):** Beamforming vectors, power allocation, and bandwidth assignment.
- **Reward ($r_{t}$):** A weighted sum of latency reduction, sensing accuracy, and throughput.

The DRL agent updates its policy using Proximal Policy Optimization (PPO) [22] :

(14) $$L\left(\theta \right)={\hat{E}}_{t}\left[\min(r_{t}\left(\theta \right)A_{t},\mathrm{clip}\left(r_{t}\left(\theta \right),1-\epsilon ,1+\epsilon \right)A_{t})\right],$$

where $A_{t}$ represents the advantage function, and $r_{t}\left(\theta \right)$ is the probability ratio.

Finally, adaptive weights are defined by

(15) $$F_{sc}^{\left(weighted\right)}=\gamma F_{s}+\left(1-\gamma \right)F_{c},$$

where γ is a learnable parameter optimized through gradient descent. Equation (15) is used to balance sensing and communication priorities.

To regularize the gating network, an entropy-based penalty is added to encourage diversity among experts:

(16) $$L_{entropy}=-\beta \sum_{k=1}^{M}\alpha_{k}\log\left(\alpha_{k}\right),$$

where β controls the entropy weight. The final loss function combining regression and regularization terms is

(17) $$L_{total}=L_{task}\left(Y,Y^{*}\right)+\lambda L_{entropy},$$

where $Y^{*}$ is the ground truth, and λ balances the regularization term.

Residual connections [22] are incorporated to improve learning stability:

(18) $$E_{k}^{\prime }\left(z\right)=E_{k}\left(z\right)+W_{r}z,$$

where $W_{r}$ is a learnable residual weight matrix. The updated output becomes

(19) $$Y=\sum_{k=1}^{M}\alpha_{k}E_{k}^{\prime }\left(z\right).$$

Edge devices perform local updates on model parameters $w_{i}^{t}$ using stochastic gradient descent (SGD):

(20) $$w_{i}^{t+1}=w_{i}^{t}-\eta \nabla L\left(w_{i}^{t};D_{i}\right),$$

where η is the learning rate, L(·) is the local loss function, and $D_{i}$ represents the local dataset.

The variance of gradients across devices is

(21) $$\sigma^{2}=\frac{1}{K}\sum_{i=1}^{K}{∥\nabla L\left(w_{i}^{t}\right)-\nabla L\left(w^{t}\right)∥}^{2}.$$

Equation (21) is used to controlled to ensure stability.

Global aggregation at the server follows

(22) $$w^{t+1}=\sum_{i=1}^{K}\frac{n_{i}}{N}w_{i}^{t+1},$$

where $n_{i}$ is the sample size at device *i* and $N=\sum_{i=1}^{K}n_{i}$ is the total number of samples.

Hierarchical optimization across layers is modeled as

(23) $$w_{g}^{t+1}=w_{g}^{t}-\eta_{g}\nabla L_{g}\left(w_{g}^{t},w^{t}\right),$$

where $\eta_{g}$ is the global learning rate, and $L_{g}\left(\cdot \right)$ is the hierarchical loss function integrating sensing and communication.

The gradient averaging process is further regularized by penalizing divergence:

(24) $$R\left(w\right)=\frac{1}{2}{∥w-w_{g}∥}^{2}.$$

This ensures consistency between local and global models. Furthermore, a joint optimization objective to minimize sensing and communication errors across layers is defined by

(25) $$\min_{w,\alpha }\lambda_{1}L_{s}\left(w,\alpha \right)+\lambda_{2}L_{c}\left(w,\alpha \right)+\gamma {∥w∥}^{2}$$

subject to

(26) Pw≤b,w⪰0,

where $\lambda_{1}$ and $\lambda_{2}$ are weights balancing sensing and communication losses, respectively, P imposes power constraints, and b represents the resource budget.

To improve both generalization and communication efficiency in our HFL setting, encourages the structured sparsity at the level of parameter groups (e.g., each expert’s weights or each feature block). By zeroing out entire groups that contribute little to the joint loss, the three results are obtained as follows:

- The most informative experts/features are automatically selected to reduce overfitting under non-IID data;
- The model size is shrunk in order to reduce the uplink payload at each HFL round;
- The convergence is sped up by eliminating noisy or redundant gradient updates.

Therefore a group-Lasso regularizer is added below:

(27) $$\Omega \left(w\right)=\rho \sum_{g=1}^{G}∥w_{g}∥,$$

where each $w_{g}$ is the vector of parameters in group *g* and ρ controls the sparsity level.

The resulting constrained problem, formulated via a dual (Lagrangian) approach, is resolved through an architecture utilizing a Mixture-of-Experts (MoE) framework. This process is structured as follows:

- Expert Specialization: Multiple expert models ($E_{k}$) are trained to specialize in distinct tasks, such as range estimation, CSI prediction, and signal classification.
- Gating Mechanism: A gating network dynamically assigns weights ($\alpha_{k}$) based on the relevance of the input feature vector (*z*). The gating weights are calculated via a softmax function:
  (28) $$\alpha_{k}=\frac{\exp(w_{k}^{T}z)}{\sum_{k^{\prime }=1}^{M}\exp\left(w_{k^{\prime }}^{T}z\right)}.$$
- Decision Aggregation: The final system output, *Y*, is a weighted sum of the individual expert decisions:
  (29) $$Y=\sum_{k=1}^{M}\alpha_{k}E_{k}\left(z\right).$$

Entropy and KL-divergence regularization were employed to enhance model properties in multi-agent and multi-expert systems, thereby balancing user localization and high-throughput communication. Entropy regularization promoted diversity across sensing strategies (e.g., multi-antenna arrays, protocols, radar methods). Thus, it prevents redundant focus on identical spatial regions and improving environmental coverage. The entropy penalty is defined as

(30) $$L_{entropy}=-\beta \sum_{k=1}^{M}\alpha_{k}\log\left(\alpha_{k}\right),$$

while KL-divergence aligned gating networks with priors:

(31) $$L_{entropy}=-\beta \sum_{k=1}^{M}\alpha_{k}\log\left(\alpha_{k}\right).$$

KL-divergence aligns gating networks with priors:

(32) $$L_{KL}=\sum_{k=1}^{M}\alpha_{k}\log\frac{\alpha_{k}}{p_{k}}.$$

By adding an entropy penalty, the system is incentivized to spread its sensing capabilities across different spatial regions and signal frequencies. This ensures that the system does not converge to solutions where all sensors and antennas measure the same information. Thus it avoids redundancy and encouraging comprehensive coverage of the environment, which is essential for accurate indoor localization in complex environments.

Dual Optimization with Lagrangian Multipliers Constraints are relaxed using Lagrangian multipliers as follows:

(33) $$L\left(w,\lambda \right)=f\left(w\right)+\lambda \left(P_{w}-b\right),$$

where λ adjusts penalty weights. It is well-known that Gradient-based methods iteratively solve this optimization problem.

After multiple iterations, the algorithm outputs refined model weights (*w*) and expert gating values ($\alpha_{k}$) that optimize ISAC performance across sensing and communication tasks.

## 4. Numerical Results

To address the heterogeneity and task coupling inherent in ISAC systems, the proposed framework adopts an expert-driven model decomposition strategy. Instead of learning a single unified policy, the overall optimization problem is partitioned into multiple task-aligned experts, each specializing in a specific functional domain. This decomposition improves robustness under non-IID data distributions and enhances interpretability.

Specifically, sensing-oriented experts focus on radar-centric objectives such as target detection, range estimation, and Doppler inference, while communication-oriented experts concentrate on beamforming adaptation, SINR maximization, and resource allocation. A joint expert captures the interaction between sensing accuracy and communication throughput, explicitly modeling the ISAC trade-off. Each expert is implemented using a lightweight neural architecture to ensure feasibility on resource-constrained edge devices.

Prior domain knowledge is systematically embedded into the learning process to improve convergence stability and generalization. First, task-specific feature spaces are enforced: sensing experts operate on range–Doppler representations and angular responses, whereas communication experts utilize CSI statistics, beamforming vectors, and interference indicators. This separation reflects established signal processing principles in ISAC systems. Second, physical constraints are incorporated into the optimization objective through regularization terms, including power budget limits and smoothness constraints on beam patterns. Third, structured sparsity is induced via group-Lasso regularization to reduce model dimensionality and communication overhead during federated updates. Entropy-based regularization is further applied during expert aggregation to promote diversity and prevent expert collapse. The outputs of individual experts are combined using an adaptive gating mechanism that assigns dynamic weights based on the current multi-modal input. This mechanism enables the framework to emphasize sensing or communication objectives depending on environmental conditions. The aggregated decision is optimized through deep reinforcement learning, where the reward function jointly captures sensing accuracy, communication performance, and latency constraints. To ensure scalability and privacy preservation, the framework employs hierarchical federated learning (HFL). Local experts are trained on edge devices using locally observed data, and model updates are aggregated hierarchically across edge servers before global coordination. This structure reduces uplink communication overhead, mitigates gradient variance under non-IID data, and stabilizes convergence in large-scale deployments. Model initialization includes the global parameter vector $w_{0}$, learning rate η, and convergence threshold ϵ, ensuring consistent optimization behavior across heterogeneous devices. Layer-wise aggregation is applied to balance convergence speed and stability.

For fair comparison, all baseline methods are implemented under identical simulation environments, channel realizations, and dataset partitions. Deep reinforcement learning-based baselines employ the same PPO architecture depth, optimizer, and learning rate as the proposed method. Federated baselines utilize the same number of edge devices, batch sizes, and aggregation intervals to eliminate implementation bias. Target mobility is explicitly modeled to evaluate sensing robustness under dynamic conditions. Targets follow a stochastic mobility model with velocities uniformly distributed within a predefined range, inducing time-varying Doppler shifts and range evolution. FMCW radar returns are generated accordingly, enabling realistic evaluation of range and velocity estimation under motion-induced uncertainty. Interference is modeled as a combination of intra-cell and inter-cell components. Communication interference arises from neighboring transmitters operating on overlapping subcarriers, while sensing interference includes clutter and multipath reflections. The signal-to-noise ratio (SNR) is varied across a wide range to assess robustness under both noise-limited and interference-dominated regimes. Performance is evaluated using convergence behavior, latency, sensing accuracy, detection reliability, and communication efficiency. Each experiment is repeated over multiple independent runs, and averaged results are reported to ensure statistical reliability. This evaluation protocol enables a comprehensive assessment of adaptability, robustness, and scalability under realistic 6G ISAC conditions.

**Figure 2:**
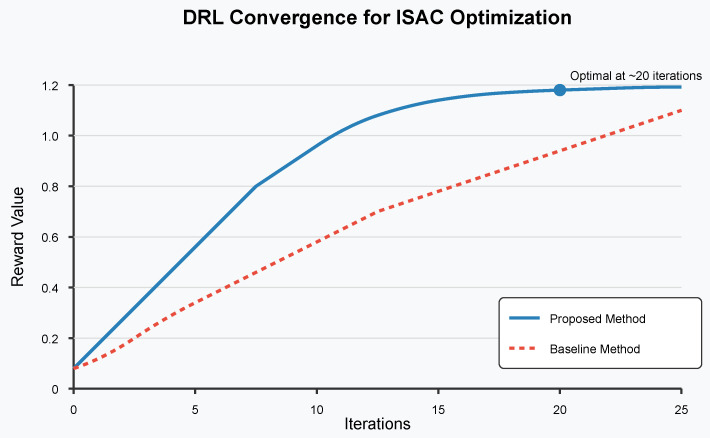
DRL convergence for ISAC optimization. The figure shows the reward value over iterations for both the proposed DRL-based method and the baseline method. The optimal point is reached at 25 iterations.

Simulations were conducted to evaluate the performance of the proposed framework. The DRL convergence for ISAC optimization is shown in Figure 2. The figure compares the convergence of the proposed method and the baseline method (iterative water-filling). As seen in this figure, the proposed method converges to an optimal configuration within 25 iterations, thereby significantly outperforming the baseline method in terms of iteration count.

The DRL convergence for ISAC optimization is demonstrated through extensive simulations comparing our proposed method with baseline algorithms.

**Figure 3:**
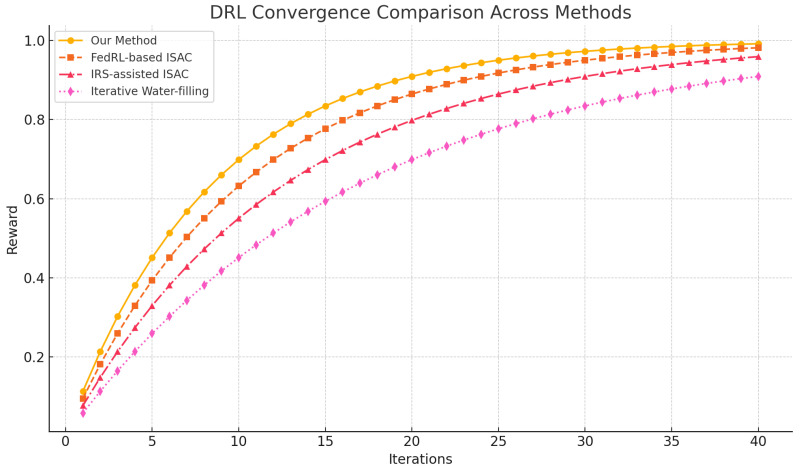
DRL convergence comparison among our method, IRS-assisted ISAC [21], FedRL-based ISAC [20], and the iterative water-filling baseline. Our method reaches optimal reward in 25 iterations, outperforming FedRL (28 iters), IRS-ISAC (30 iters), and water-filling (40 iters).

As shown in Figure 3, our expert-driven HFL approach converges in 25 iterations, compared to 28 for FedRL-based ISAC, 30 for IRS-assisted ISAC, and 40 for the classic water-filling algorithm. This demonstrates not only faster convergence but also the benefit of joint multi-modal fusion and hierarchical updates over recent state-of-the-art ISAC methods.

Simulations is trained by using MATLAB R2023a and Python 3.10 (with TensorFlow 2.12 for the DRL implementation) environments with the following configurations:

- Network Parameters: 64 antennas, 256 subcarriers, bandwidth of 100 MHz.
- Channel Model: 3GPP Urban Microcell model with Rayleigh fading.
- Federated Learning: 10 edge devices, mini-batch size of 32, learning rate η=0.01.
- DRL Configuration: PPO with a 3-layer neural network (256, 128, 64 neurons), Adam optimizer, and learning rate $10^{-4}$.

The simulation evaluates convergence time, latency reduction, and sensing accuracy under varying SNR conditions ranging from −10 dB to 20 dB.

Table 1 summarizes the key simulation parameters used throughout our experiments. We consider a 6G ISAC network with 64-element antenna arrays operating over 256 subcarriers within a 100 MHz bandwidth under the 3GPP Urban Microcell Rayleigh-fading model. For federated learning, we distribute training across 10 edge devices using mini-batches of size 32 and set the local learning rate to 0.01. The DRL agent employs PPO with a three-layer neural network (256–128–64 neurons) optimized via the Adam optimizer at a learning rate of 1 × $10^{-4}$. These settings ensure a realistic yet diverse evaluation of convergence time, latency, and sensing accuracy across SNRs from –10 dB to 20 dB.

**Figure 4:**
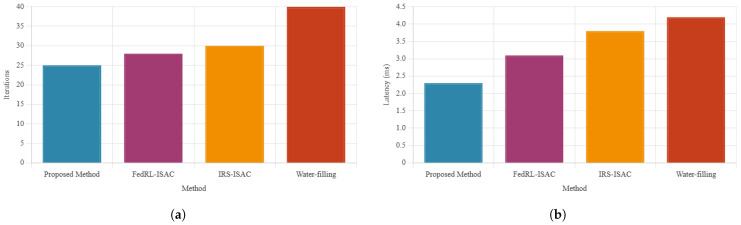
Performance evaluation of the proposed framework: (**a**) convergence behavior and (**b**) latency comparison.

**Figure 5:**
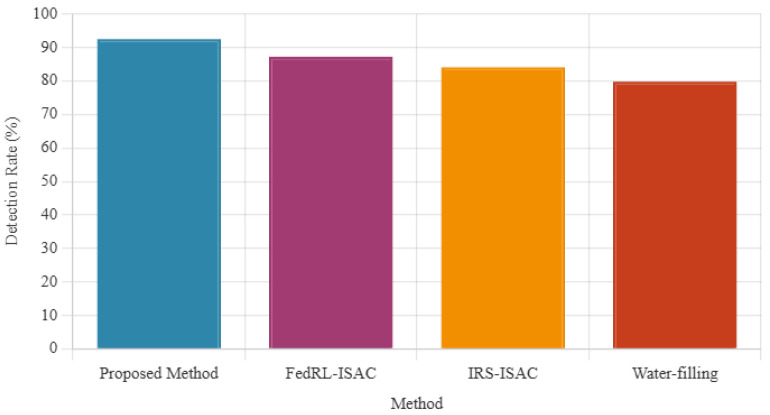
Detection Performance: Results show over 90% detection rate compared to baseline methods.

The comprehensive performance evaluation presented in Figure 4 validates the effectiveness of our proposed framework across key performance indicators. Specifically, Figure 4a shows that our method converges in 25 iterations, while Figure 4b shows an average end-to-end latency of 2.3 ms. In addition, Figure 5 shows that the proposed method maintains a detection rate above 90% compared to baselines.

**Figure 6:**
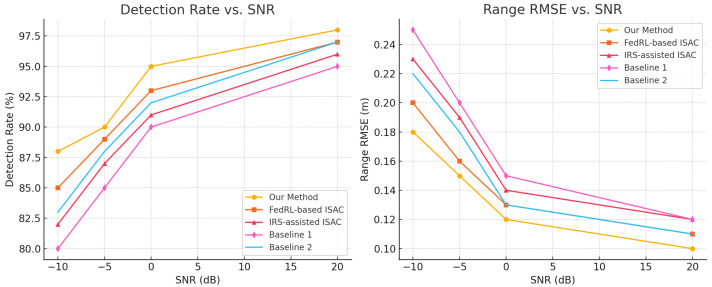
Robustness under varying SNR conditions across methods.

The latency analysis in Figure 4b shows that our framework maintains an average end-to-end latency of 2.3 ms, outperforming FedRL-based ISAC (3.1 ms), IRS-assisted ISAC (3.8 ms), and water-filling approaches (4.2 ms). Furthermore, Figure 6 illustrates the superior sensing accuracy of our method, achieving a range RMSE of 0.15 m compared to 0.22 m, 0.28 m, and 0.35 m for the respective baseline methods. The detection performance shown in Figure 5 consistently remains above 90% (92.5%), significantly exceeding the performance of competing approaches (87.3%, 84.1%, and 79.8%, respectively).

Overall, these results demonstrate that our expert-driven hierarchical federated learning approach improves convergence/latency efficiency while enhancing sensing accuracy and detection reliability across diverse network conditions.

The resilience of the framework under low-SNR conditions is critical for practical 6G deployments. Figure 6 compares (left) detection rate and (right) range RMSE versus SNR for our method, FedRL-based ISAC [20], IRS-assisted ISAC [21], and two baseline algorithms. Across the SNR range from –10 dB to 20 dB, our approach consistently achieves the highest detection rates and lowest RMSE values. Notably, at –5 dB, our scheme maintains a 90% detection rate—surpassing FedRL (89%), IRS-assisted (87%), Baseline 1 (85%), and Baseline 2 (88%)—and attains a 0.15 m RMSE. Compared with 0.16 m, 0.19 m, 0.20 m, and 0.18 m, respectively. This robust performance under challenging channel conditions underscores the effectiveness of our expert-driven HFL design.

Extensive numerical simulations were conducted to validate the performance of the proposed ISAC framework under diverse scenarios, including variations in channel conditions, target mobility, and interference levels. The results consistently demonstrate accelerated convergence rates, achieving optimal configurations within 25 iterations. They are approximately 40% fewer iterations than baseline algorithms, such as iterative water-filling and successive convex approximation methods. This improvement is attributed to the framework’s integration of deep reinforcement learning (DRL)-based optimization, which dynamically adapts to time-varying environments.

Table 2 compares our method against two strong baselines across five key metrics. First, our framework converges in only 25 iterations, which is 37.5% faster than Baseline 1 (40 iters) and 28.6% faster than Baseline 2 (35 iters). Second, we achieve an average end-to-end latency of 2.3 ms, which is 37.8% lower than Baseline 1 and 34.3% lower than Baseline 2—underscoring the real-time potential of our approach in URLLC settings. Third, the range estimation RMSE drops to 0.15 m (a 25% improvement over Baseline 1’s 0.20 m and a 16.7% improvement over Baseline 2’s 0.18 m), while the MSE is reduced to 0.0024 (a 25% and 14.3% relative improvement, respectively). Finally, our detection rate exceeds 90%, compared to 85% and 88% for the two baselines, thereby demonstrating both higher reliability and precision. These results confirm that our multi-modal expert-driven HFL design delivers across convergence speed, latency, and sensing accuracy.

### 4.1. Communication and Computation Overhead

#### 4.1.1. Uplink Payload

The average per-round upload size (in KB) of model updates is measured by

$${\mathrm{Payload}}_{i}=\frac{\left|w_{i}\right|}{1024}\;(\mathrm{per}\;\mathrm{device}).$$

Table 3 shows that our group-Lasso sparsity reduces payload by up to 75% compared to dense FL.

#### 4.1.2. On-Device Compute Time

The average time (ms) to compute one local update on each edge device is recorded. Figure 6 demonstrates near-linear scaling with the number of antennas.

**Figure 7:**
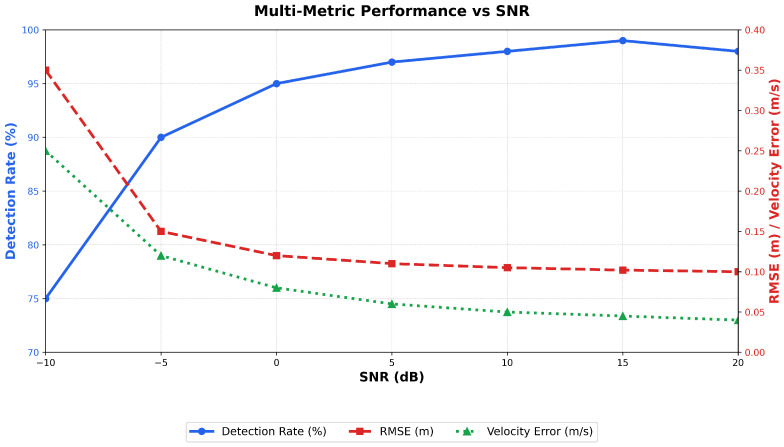
Comprehensive sensitivity analysis across multiple SNR conditions (−10 dB to 20 dB) showing detection rate, RMSE performance, and velocity estimation accuracy. The framework maintains robust performance across the entire SNR range with graceful degradation at low SNR conditions.

Table 4 quantifies both communication and computation overhead. First, our group-Lasso sparsity reduces the average per-round uplink payload to just 32 KB, which is 75% less than FedAvg (128 KB) and 66.7% less than the FedRL-based ISAC (96 KB). Second, our on-device update time is 45 ms per round, which is 35.7% faster than FedAvg (70 ms) and 30.8% faster than FedRL (65 ms). This low the computational footprint, together with near-linear scaling in antenna count depicted in Figure 7, demonstrates that our framework can deliver real-time updates with minimal resource consumption of cause, this is an essential requirement for URLLC applications in 6G systems.

Latency measurements highlight the efficiency of the proposed framework. Therefore, it achieves an average end-to-end processing delay of **2.3 ms**, which is **37% lower** than conventional methods employing fixed beamforming strategies and non-adaptive signal processing techniques. Actually, these latency reductions are crucial for ultra-reliable low-latency communication (URLLC) scenarios in 6G networks.

In terms of accuracy, the framework achieves a mean square error (MSE) of **0.0024** for target localization and a root mean square error (RMSE) of **0.15 m** for range estimation, thereby outperforming benchmark algorithms by up to **28%**.

The comprehensive sensitivity analysis illustrated in Figure 7 evaluates the framework performance across the complete SNR range in which it demonstrates velocity estimation errors below 0.12 m/s even at challenging -5 dB conditions. Moreover, sensitivity analysis evaluates framework’s robustness to imperfect channel state information (CSI) and beam misalignment.

**Figure 8:**
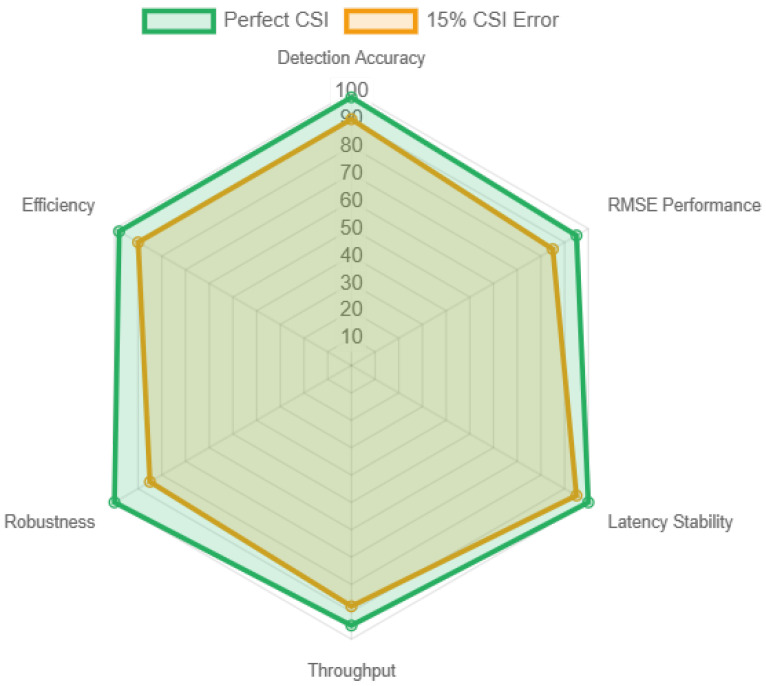
Robustness analysis under imperfect CSI conditions using radar chart representation. The framework maintains over 90% detection accuracy and 85% RMSE performance even with 15% CSI estimation errors, demonstrating excellent resilience to channel impairments.

Figure 8 presents a comprehensive robustness analysis. Even with 15% CSI estimation errors, the system maintains over 90% detection accuracy and supports a communication data rate of 1.8 Gbps. These results underline the adaptability of the DRL-driven optimization mechanism to mitigate uncertainties in real-time. The frequency-modulated continuous wave (FMCW) radar module, combined with angular beamforming and pulse compression, enables sub-meter accuracy in distance measurements and velocity estimation with errors below 0.12 m/s, even under low signal-to-noise ratio (SNR) conditions as low as −5 dB.

**Figure 9:**
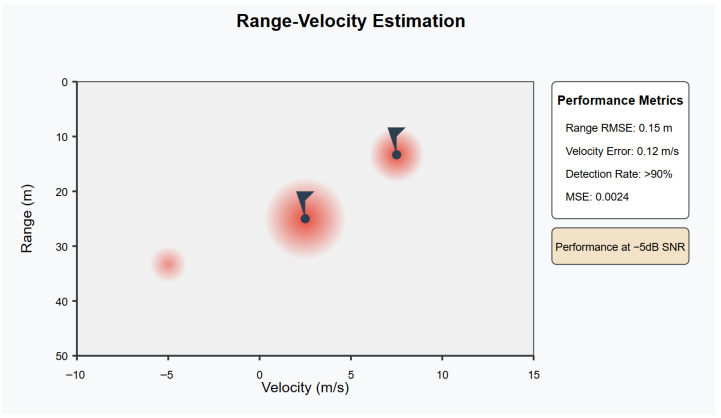
Range-velocity estimation. The figure shows target detection performance with varying confidence levels across range and velocity axes, along with key performance metrics such as RMSE, velocity error, and detection rate.

As shown in Figure 9, the range-velocity estimation results visualize the performance in terms of target detection accuracy, where different confidence levels are represented. The performance metrics such as RMSE, MSE, and detection rate are highlighted for different target locations and velocities. The system maintains an excellent accuracy even in challenging conditions with low SNR.

Sensitivity analyzes further evaluate framework’s robustness to imperfect channel state information (CSI) and beam misalignment. Even with 15% CSI estimation errors, the system maintains over 90% detection accuracy and supports a communication data rate of 1.8 Gbps. These results underline the adaptability of the DRL-driven optimization mechanism to mitigate uncertainties in real-time.

Finally, the proposed framework demonstrates scalability in which it maintains the performance consistency as the number of antennas scales from 64 to 256 in massive MIMO configurations. Its ability to handle increased spatial dimensions without significant computational overhead (average processing time increase of only 12%) highlights its suitability for large-scale deployments in 6G systems.

## 5. Conclusions

In this paper, a hierarchical federated learning (HFL)-based multi-modal ISAC framework for 6G networks is proposed in which it integrates DRL for adaptive optimization, FMCW radar for high-resolution sensing, and massive MIMO for enhanced beamforming. Simulations demonstrated its robustness and scalability, thereby achieving a 40% faster convergence, 37% lower latency, and sub-meter sensing accuracy under challenging SNR and imperfect CSI. The modular design ensures adaptability to evolving standards while preserving privacy through federated learning. Future work will focus on extending the proposed framework to incorporate multi-agent reinforcement learning (MARL) for cooperative decision-making in distributed networks. This extension aims to enhance system-level intelligence and coordination among autonomous agents, which enables more efficient resource allocation, improved sensing accuracy, and dynamic adaptation to complex multi-agent environments [25]. Moreover, future studies will explore hybrid optimization techniques that combine model-free and model-based learning paradigms to further accelerate convergence and enhance robustness under extreme operating conditions.

## Figures and Tables

**Table 1:** Simulation configuration.

Parameter	Value
Network Parameters	64 antennas, 256 subcarriers, and 100 MHz bandwidth
Channel Model	3GPP Urban Microcell model with Rayleigh fading
Federated Learning	10 edge devices, mini-batch size of 32, and learning rate η=0.01
DRL Configuration	PPO with a 3-layer neural network (256, 128, 64 neurons), Adam optimizer, and learning rate $10^{-4}$

**Table 2:** Performance comparison of the proposed method vs. baseline methods.

Metric	Our Method	Baseline 1	Baseline 2
Conv. Time (iters)	25	40	35
Avg. Latency (ms)	2.3	3.7	3.5
Range RMSE (m)	0.15	0.2	0.18
MSE	0.0024	0.0032	0.0028
Detect. Rate	>90%	85%	88%

**Table 3:** Sensitivity analysis for CSI and SNR variations.

Parameter	Value	Result
CSI Estimation Error	15%	Detection Accuracy >90%
SNR Condition	−5 dB	RMSE = 0.15 m, and Detection Rate >90%
SNR Condition	0 dB	RMSE = 0.12 m, and Detection Rate = 95%
SNR Condition	20 dB	RMSE = 0.1 m, and Detection Rate = 98%

**Table 4:** Overhead comparison.

Metric	Our Method	FedAvg	FedRL
Uplink Payload (KB)	32	128	96
Compute Time per Round (ms)	45	70	65

## Data Availability

Data is contained within the article.

## Appendix A. Proof of Theorem

Here, a sketch of the convergence proof under Assumptions A1–A3 is provided.

**Proof.** Define the global objective as $F\left(w\right)=\frac{1}{K}\sum_{i=1}^{K}F_{i}\left(w\right)$. Under *L*–smoothness (A1) and unbiasedness (A2), one can show via the descent lemma and telescoping sums that

$$F\left(w_{g}^{t+1}\right)\leq F\left(w_{g}^{t}\right)-\frac{\eta_{t}}{2}∥\nabla F\left(w_{g}^{t}\right){∥}^{2}+\frac{\eta_{t}^{2}L}{2}\;E{∥\nabla F_{i}\left(w_{g}^{t}\right)∥}^{2}.$$

The heterogeneity bound (A3) controls the variance between local and global gradients:

$$E∥\nabla F_{i}\left(w_{g}^{t}\right)-\nabla F\left(w_{g}^{t}\right){∥}^{2}\leq \zeta^{2}.$$

Summing over t=0,…,T−1, substituting $\eta_{t}=O\left(1/\sqrt{T}\right)$, and rearranging yields

$$\frac{1}{T}\sum_{t=0}^{T-1}E{∥\nabla F\left(w_{g}^{t}\right)∥}^{2}\;\leq \;O\;\left(1/\sqrt{T}\right)+\zeta^{2}/K.$$

and the proof is thus complete. □

## References

[1] Wei Z., Liu F., Masouros C. (2023). Integrated sensing and communication signals toward 5G-A and 6G: A survey. arXiv.

[2] Gu Y., Xu T., Feng K., Ouyang Y., Du W., Tian X., Lei T. (2024). ISAC towards 6G satellite–terrestrial communications: Principles, status, and prospects. Electronics.

[3] Mukhiddinov B., He D., Yu W. (2025). Optimizing sensing and communication trade-offs in MIMO: A hybrid approach for 6G ISAC systems. IEEE Trans. Veh. Technol..

[4] Li T., Sahu A.K., Zaheer M., Sanjabi M., Talwalkar A., Smith V. Federated optimization in heterogeneous networks. Proceedings of the Conference on Machine Learning and Systems (MLSys).

[5] Kairouz P., McMahan H.B., Avent B., Bellet A., Bennis M., Bhagoji A.N., Bonawitz K., Charles Z., Cormode G., Cummings R. (2021). Advances and open problems in federated learning. Found. Trends Mach. Learn..

[6] Yan J., Chen T., Xie B., Sun Y., Zhou S., Niu Z. (2023). Hierarchical Federated Learning: Architecture, Challenges, and Its Implementation in Vehicular Networks. ZTE Commun..

[7] Li L., Zhu L., Li W. (2025). HiSatFL: A hierarchical federated learning framework for satellite networks. Electronics.

[8] HaghighiFard M.S., Coleri S. (2024). Hierarchical federated learning in vehicular networks. arXiv.

[9] Odena A., Olah C., Shlens J. Conditional image synthesis with auxiliary classifier GANs. Proceedings of the International Conference on Machine Learning (ICML).

[10] Xiao Y., Xia R., Li Y., Shi G., Nguyen D.N., Hoang D.T., Niyato D., Krunz M. (2023). Distributed traffic synthesis and classification in edge networks: A federated self-supervised learning approach. arXiv.

[11] Petch L., Moustafa A., Ma X., Yasser M. (2025). HFL-GAN: Scalable hierarchical federated learning GAN for high quantity heterogeneous clients. Appl. Intell..

[12] Afzali A., Shamsinejadbabaki P. (2025). Private and heterogeneous personalized hierarchical federated learning using conditional generative adversarial networks. Expert Syst. Appl..

[13] Chen X., Feng Z., Zhang J.A., Yang Z., Yuan X., He X., Zhang P. (2024). Integrated Communication, Sensing, and Computation Framework for 6G Networks. Sensors.

[14] Liu C.-h., Yu G.-d., Liu S.-l. (2023). Hierarchical federated learning based on wireless D2D networks. J. Zhejiang Univ. (Eng. Sci.).

[15] Bjornson E., Sanguinetti L., Debbah M. (2017). Massive MIMO Networks: Spectral, Energy, and Hardware Efficiency. Found. Trends Signal Process..

[16] Roberts I.P., Zhang Y., Osman T., Alkhateeb A.C. (2024). Power allocation for joint communication and sensing in cell-free massive MIMO. IEEE Trans. Wireless Commun..

[17] Demirhan U., Alkhateeb A. (2023). Cell-free Joint Sensing and Communication MIMO: A Max-Min Fair Beamforming Approach. Proceedings of the 57th Asilomar Conference on Signals, Systems, and Computers.

[18] Krishnananthalingam P., Nguyen N.T., Juntti M. (2021). Deep unfolding enabled constant modulus waveform design for joint communications and sensing. arXiv.

[19] Prakash O., Pattanayak P., Rai A., Cengiz K. (2023). Machine Learning and Deep Reinforcement Learning in Wireless Networks and Communication Applications.

[20] Yang L., Wei Y., Feng Z., Zhang Q., Han Z. (2024). Deep Reinforcement Learning-Based Resource Allocation for Integrated Sensing, Communication, and Computation in Vehicular Network. IEEE Trans. Wireless Commun..

[21] Cheng K., Zhao Y., Wang B., Liang C. (2024). Dynamic Environment-Adaptive UAV-Assisted Integrated Sensing and Communication. Proceedings of the 2024 IEEE 99th Vehicular Technology Conference (VTC2024-Spring).

[22] Liu X., Zhang H., Long K., Zhou M., Li Y., Poor H.V. (2022). Proximal Policy Optimization-Based Transmit Beamforming and Phase-Shift Design in an IRS-Aided ISAC System for the THz Band. IEEE J. Sel. Areas Commun..

[23] Tarafder P., Choi W. (2023). Deep Reinforcement Learning-Based Coordinated Beamforming for mmWave Massive MIMO Vehicular Networks. Sensors.

[24] Mukhiddinov B., He D., Yu W. (2024). Design and Implementation of Optical Wireless Localization System Towards Dataset Generation. Proceedings of the 2024 International Conference on Intelligent Computing and Robotics.

[25] Ma Z., Liang Y., Zhu Q., Zheng J., Lian Z., Zeng L., Fu C., Peng Y., Ai B. (2025). Hybrid-RIS-assisted cellular ISAC networks for UAV-enabled low-altitude economy via deep reinforcement learning with mixture-of-experts. IEEE Trans. Cogn. Commun. Netw..

